# Focus of infection and microbiological etiology in community-acquired infections in hospitalized adult patients in the Faroe Islands

**DOI:** 10.1186/s12879-018-3650-3

**Published:** 2019-01-07

**Authors:** Marija Todorovic Markovic, Court Pedersen, Magnús Gottfredsson, Mirjana Todorovic Mitic, Shahin Gaini

**Affiliations:** 1 Medical Department, Infectious Diseases Division, National Hospital of the Faroe Islands, JC. Svabosgøta 41-49, Tórshavn, Faroe Islands; 2 Department of Infectious Diseases, Odense University Hospital and University of Southern Denmark, Odense, Denmark; 3 Department of Infectious Diseases, Landspitali University Hospital, Reykjavík, Iceland; 4 Faculty of Medicine, School of Health Sciences, University of Iceland, Reykjavik, Iceland; 5 Clinic of Oncology, Clinical Centre, Nis, Serbia; 6 Centre of Health Research and Department of Science and Technology, University of the Faroe Islands, Torshavn, Faroe Islands

**Keywords:** Community-acquired infection, Sepsis, Etiology, Focus of infection

## Abstract

**Background:**

The aim of the present study was to gain national data on the clinical and microbiological characteristics of community-acquired infections in the Faroe Islands and to compare these data with data from other geographical areas.

**Methods:**

A prospective, observational study involving all patients > = 16 years admitted at the Department of Medicine at the National Hospital, Torshavn, Faroe Islands from October 2013 until April 2015.

**Results:**

Of 5279 admissions, 1054 cases were with community-acquired infection and were included in the study. Out of these 1054 cases, 471 did not meet the criteria for SIRS (Systemic Inflammatory Response Syndrome), while the remaining 583 cases had sepsis. Mean age was 68 years. At least one comorbidity was found in 80% of all cases. Documented infections were present in 75%, and a plausible pathogen was identified in 29% of all cases. The most common gram-positive pathogen was *Staphylococcus aureus,* and the most frequent gram-negative pathogen was *Escherichia coli*. The most common focus of infection was lower respiratory tract, followed by urinary tract, and skin-soft tissue/bone-joint. Bacteremia was found in 10% of the cases.

**Conclusion:**

In community-acquired infections in hospitalized patients in the Faroe Islands the lower respiratory tract and the urinary tract were the most frequent foci of infection. Gram-negative pathogens and *Escherichia coli* were the most frequent pathogens in infection without Systemic Inflammatory Response Syndrome, in sepsis and in bacteremia. Our data on clinical characteristics and microbiological etiology provide new information which may be used to develop local guidelines for the managing of patients admitted with community-acquired infections.

**Electronic supplementary material:**

The online version of this article (10.1186/s12879-018-3650-3) contains supplementary material, which is available to authorized users.

## Background

Infections carry significant morbidity and mortality worldwide [1, 2]. Extensive research has been done in relation to infections among patients admitted to hospital, but often the focus has been on more severe forms of infection, or on specific sites such as pneumonia or urinary tract infections. According to de Prost et al. only 40–60% of severe sepsis or septic shock cases have a microbiologically confirmed infection [3]. These authors argue that this is due to antibiotic therapy received prior to the onset of organ dysfunction, insufficient or incomplete diagnostic workup, or the presence of unusual organisms that are difficult to identify in routine practice. Some infections have even lower percentage of microbiological confirmation [4–7].

The progression from onset of infection to sepsis can be insidious and unpredictable [8]. Therefore, proper diagnosis and treatment in the early stages of infections are essential to the outcome [9].

The pattern of infectious diseases may vary from country to country. Therefore, regional research regarding different aspects of community-acquired infection such as incidence, microbial etiology and focus of infection is essential for understanding the burden of infection locally in community, and for developing regional and national strategies for diagnosing and treating infectious diseases. Although research has been done on specific infections in the Faroe Islands, no studies have been done describing the general characteristics of community-acquired infections in the country. The aim of the present study was to gain data on the clinical characteristics of community-acquired infections in the Faroe Islands at the present time, and to compare these data with data from other geographical areas.

## Methods

### Study design and setting

This study is based on a prospective observational epidemiological study on sepsis in medical patients in the Faroe Islands. A previous publication on the epidemiological aspects of this cohort has been published [10]. More detailed information on the methodology is presented in the previous published paper [10]. In short, all medical patients admitted at the Department of Medicine at the National Hospital of the Faroe Islands were included in the study in the period from October 1st, 2013 to April 1st, 2015 [10]. The National Hospital of the Faroe Islands is the central hospital in the country, serving 37870 inhabitants in its catchment area in the study period [11]. This corresponds to 80% of the population of the Faroe Islands [11]. All medical adult patients admitted at the Department of Medicine at the National Hospital of the Faroe Islands in the study period were included in the study [10].

### Patient selection

All medical patients > = 16 years of age admitted at the Department of Medicine or at the Intensive Care Unit at the National Hospital of the Faroe Islands in the study period were included.

All included patients were investigated in detail regarding signs and symptoms of infection occurring within the first 48 h of admission [10]. Patients with hospital-acquired infections were excluded from the study [10]. Patients transferred to the Department of Medicine either from surgical departments or from two other Faroese hospitals were also excluded from the study [10].

All data were collected in a prospective manner. Vital signs and laboratory data were collected in the first 48 h of admission. Patients were classified in a group of infected patients and in a group of patients without infection by using pre-study defined consensus criteria (Additional file 1). The focus of infection was characterized and the Systemic Inflammatory Response Syndrome (SIRS) criteria were used for the sepsis classification [12]. Sepsis severity was characterized with the Sepsis-related Organ Failure Assessment (SOFA) Score with oxygen saturation as a variable for respiratory failure [13] (Appendix 1).

Results from blood cultures and other microbiological specimens (cerebrospinal fluid, sputum/secretions from the respiratory tract, urine, abscess drainage, feces) were registered from all included patients. Only samples obtained in the first 48 h of admission were used. Blood cultures were done using two aerobic and two anaerobic bottles (BD BACTEC, Benex Limited, Dun Laoghaire, Ireland). Blood cultures with possible contamination were excluded from the analysis. Coagulase-negative staphylococci, *Corynebacterium* spp., *Proprionibacterium acnes*, and *Bacillus* spp. were considered as contaminants unless they were isolated from two or more separate blood-culture sets [14].

The microbiological analyses were done locally at our Laboratory of Clinical Microbiology at the National Hospital of the Faroe Islands. Our laboratory uses standard methods for pathogen identification and resistance testing (EUCAST) used in Scandinavia and in the Nordic countries. More specialized analyses and confirmatory tests were done at the National Reference Laboratory of Clinical Microbiology in Denmark (SSI, Statens Serum Institut, Copenhagen, Denmark, https://www.ssi.dk/). Our Laboratory of Clinical Microbiology at the National Hospital of the Faroe Islands is supervised, and quality controlled by SSI in Copenhagen.

Information regarding the presence of co-morbidity in each single included infected patient was obtained from the electronic patient records. The Charlson comorbidity index was calculated in each patient [15].

### Definitions

Community-acquired infection was defined as an infection contracted outside of a health care facility or an infection present at the time of admission.

Infection was defined as the presence of a clinically relevant pathogen by microscopy/culture/polymerase chain reaction, positive serology result, pneumonia verified by chest-X ray, infection documented with other imaging techniques, positive urine dip test combined with symptoms of urinary tract infection, or as typical clinical symptoms such as for example erysipelas [16] (Additional file 1).

Systemic Inflammatory Response Syndrome (SIRS) was defined according to the consensus conference from 1992 [12].

Sepsis was defined as the presence of SIRS and a documented or suspected infection.

Severe sepsis was defined with the presence of at least one of the findings presented in Appendix 2.

Septic shock was defined as sepsis with persistent sepsis induced hypotension for more than one hour despite adequate fluid resuscitation.

Bacteremia was categorized as community-acquired if there were clinical evidence that the infection was present or incubating when the patient was admitted [17].

The study was planned in 2012 when the SIRS criteria were the official criteria used in sepsis studies [12]. The new Sepsis-3 criteria were published in 2016 [9]. Because the study was planned before 2016 we decided to follow our research protocol using the SIRS criteria to define sepsis.

### Data analyses

Descriptive statistics were used to summarize the demographics and characteristics of 1054 cases eligible for the analysis. Results are expressed as mean ± standard deviation and as frequencies and percentages. Significance testing between groups was performed using Chi square tests. Database management and calculation of descriptive statistics were performed using Access and Excel (Microsoft Corporation, Redmond, Washington).

### Ethical considerations

The Faroese Ethical Committee assessed that our study did not need approval according to Faroese law as it was register based. The study was approved by The Faroese Data Protection Agency (J. no: 13/00082–4). Gathered data were anonymized and kept on the hospital’s safe server.

## Results

**Fig. 1:**
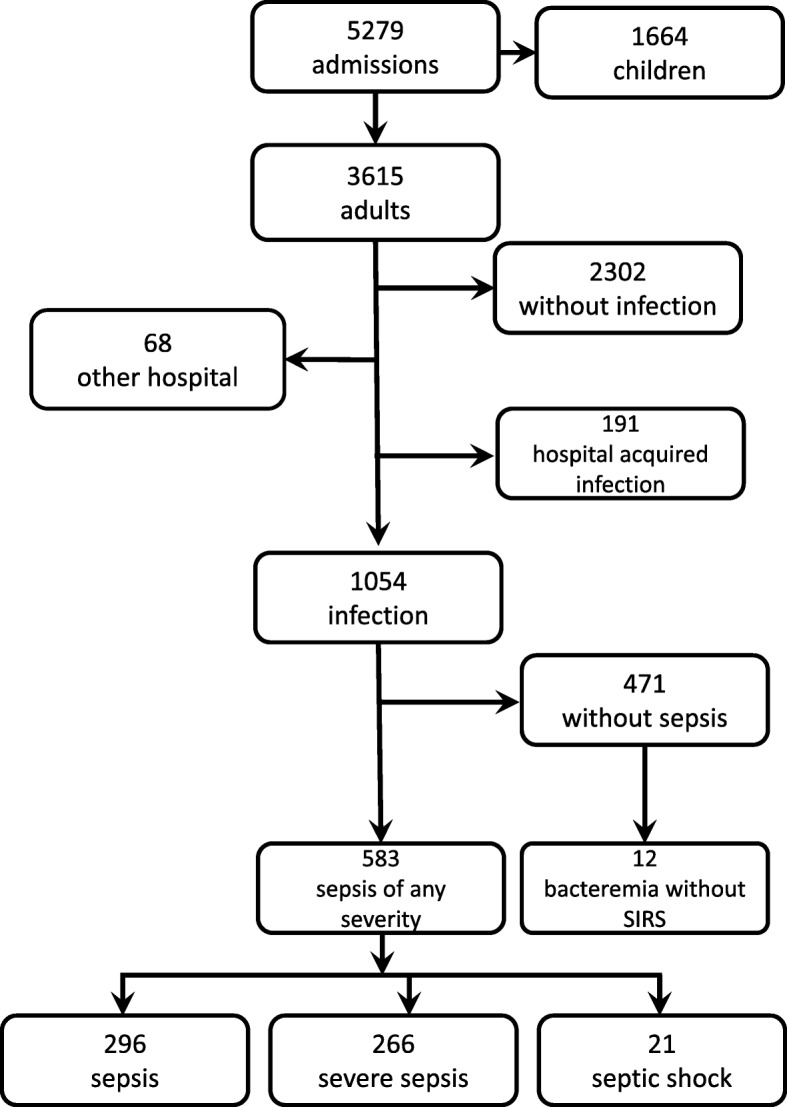
Flow diagram of enrolled patients

During the 18 months of prospective data collection, we recorded a total of 5279 admissions. Adult patients (age > = 16 years) accounted for 3615 admissions. Among these, 1054 patient admissions were associated with community-acquired infections, and these admissions were included as cases in the study. In 471 (45%) cases patients did not meet the SIRS criteria, while the remaining 583 (55%) met the definition of sepsis (Fig. 1).

Of the total study population (patients with infection), men were admitted 515 times and women 539 times. Mean age was 68 years (ranged in age from 16 to 102 years). At least one comorbidity was reported in 839 cases (80% of all cases with infection). The most common comorbidities were diabetes, chronic pulmonary disease, myocardial infarction, connective tissue diseases, cerebrovascular disease, and metastatic solid tumors. Demographic data on our study population, distribution of SIRS, qSOFA, primary sites of infection, and organ dysfunction are presented in Tables 1 and 2.

**Table 1:** Demographic characteristics of patients admitted at the Department of Medicine with infection without SIRS, sepsis, severe sepsis and septic shock in an 18-month period

Variables	Infection without SIRS	Sepsis of any severity	Sepsis	Severe sepsis	Septic shock
Total	*n* = 471	*n* = 583	*n* = 296	*n* = 266	*n* = 21
Gender, *n* (%)					
Male	217 (46.1)	298 (51.1)	141 (47.6)	145 (54.5)	12 (57.1)
Female	254 (53.9)	285 (48.9)	155 (52.4)	121 (45.5)	9 (42.9)
Age categories, yrs., *n* (%)					
15–39	50 (10.6)	56 (9.6)	44 (14.9)	11 (4.1)	1 (4.8)
40–64	92 (19.5)	142 (24.4)	68 (23.0)	69 (25.9)	5 (23.8)
65–84	241 (51.2)	291 (49.9)	159 (53.7)	124 (46.6)	8 (38.1)
85+	88 (18.7)	94 (16.1)	25 (8.4)	62 (23.3)	7 (33.3)
Charlson comorbidity index, *n* (%)					
0	92 (19.5)	123 (21.1)	68 (23.0)	51 (19.2)	4 (19.0)
1–2	176 (37.4)	213 (36.5)	104 (35.1)	102 (38.3)	7 (33.3)
> 2	203 (43.1)	247 (42.4)	124 (41.9)	113 (42.5)	10 (47.6)
Immunosuppression*, *n* (%)					
No	282 (59.9)	389 (66.7)	182 (61.5)	190 (71.4)	17 (81.0)
Yes	189 (40.1)	194 (33.3)	114 (38.5)	76 (28.6)	4 (19.0)

*Immuno-compromised state was defined by either administration in the 6 months prior to admission of steroid treatment (at least 0.3 mg/kg prednisolone), radiation therapy, chemotherapy, or as severe malnutrition, congenital immuno- humoral or cellular immune deficiency state [29]

**Table 2:** Distribution of systematic inflammatory response syndrome, infections and organ dysfunction in patients admitted at the Department of Medicine with infection without SIRS, sepsis, severe sepsis and septic shock in an 18-month period

Variables	Infection without SIRS	Sepsis of any severity	Sepsis	Severe sepsis	Septic shock
Total	*n* = 471	*n* = 583	*n* = 296	*n* = 266	*n* = 21
Bacteremia, *n* (%)					
No	459 (97.5)	496 (85.1)	265 (89.5)	216 (81.2)	15 (71.4)
Yes	12 (2.5)	87 (14.9)	31 (10.5)	50 (18.8)	6 (28.6)
No. of sources of infection per patient, *n* (%)					
1	318 (67.5)	380 (65.2)	184 (62.2)	183 (68.8)	13 (62.0)
2	21 (4.5)	57 (9.8)	19 (6.4)	35 (13.2)	3 (14.3)
3	1 (0.2)	3 (0.5)	0	3 (1.1)	0
Sites of infection, *n* (%)*					
CNS	1 (0.2)	3 (0.5)	1 (0.3)	2 (0.8)	0
Upper respiratory tract	7 (1.5)	7 (1.2)	3 (1.0)	4 (1.5)	0
Lower respiratory tract	162 (34.4)	245 (42.0)	107 (36.1)	129 (48.5)	9 (43.0)
Cardiovascular	3 (0.6)	1 (0.2)	1 (0.3)	0	0
Abdominal	15 (3.2)	16 (2.7)	5 (1.7)	9 (3.4)	2 (9.5)
Genitourinary tract	89 (18.9)	119 (20.4)	60 (20.3)	57 (21.4)	2 (9.5)
Skin-soft tissue/bone-joint	49 (10.4)	50 (8.6)	26 (8.8)	23 (8.6)	1 (4.8)
Catheter infection	15 (3.2)	31 (5.3)	6 (2.0)	22 (8.3)	3 (14.3)
Other infection	23 (4.9)	29 (5.0)	13 (4.4)	14 (5.3)	2 (9.5)
Uncertain group with bacteremia	0	10 (1.7)	5 (1.7)	5 (1.9)	0
Uncertain group without bacteremia	131 (27.8)	133 (22.8)	88 (29.7)	40 (15.0)	5 (23.8)
SIRS, *n* (%)					
Pulse rate	149 (31.6)	406 (69.6)	216 (73.0)	177 (66.5)	13 (61.9)
Temperature	75 (15.9)	296 (50.8)	164 (55.4)	126 (47.4)	6 (28.6)
Respiratory rate	12 (2.5)	427 (73.2)	211 (71.3)	198 (74.4)	18 (85.7)
Leukocyte count	73 (15.5)	368 (63.1)	187 (63.2)	170 (63.9)	11 (52.4)
SOFA positive criteria, *n* (%)					
0	183 (38.9)	148 (25.4)	131 (44.3)	17 (6.4)	0
1	181 (38.4)	183 (31.4)	115 (38.9)	68 (25.6)	0
2	53 (11.3)	126 (21.6)	40 (13.5)	83 (31.2)	3 (14.3)
3+	54 (11.5)	126 (21.6)	10 (3.4)	98 (36.8)	18 (85.7)
SIRS positive criteria, *n* (%)					
2	0	342 (58.7)	190 (64.2)	143 (53.8)	9 (42.9)
3	0	203 (34.8)	101 (34.1)	93 (35.0)	9 (42.9)
4	0	38 (6.5)	5 (1.7)	30 (11.3)	3 (14.3)
QSOFA, *n* (%)					
0	325 (69.0)	245 (42.0)	213 (72.0)	32 (12.0)	0
1	126 (26.8)	235 (40.3)	79 (26.7)	149 (56.0)	7 (33.3)
2	19 (4.0)	74 (12.7)	4 (1.4)	64 (24.1)	6 (28.6)
3	1 (0.2)	29 (5.0)	0	21 (7.9)	8 (38.1)

^*^The added number of individual sites of infection exceed the number of patients because one patient could have more than one site of infection or organ failure associated with the admission

Infections were radiologically and/or microbiologically confirmed in 780 (74%) of all cases; a plausible pathogen was identified in 304 cases (29%). Table 3 shows the breakdown of specific pathogens. A causative pathogen was identified in 169 cases (36%) of infection without SIRS, 120 cases (41%) of sepsis, 140 cases (53%) of severe sepsis and 13 cases (62%) of septic shock.

**Table 3:** Microbiological etiology in community-acquired infection

Microorganism	Infection without SIRS	Sepsis	Severe sepsis	Septic shock
Gram-positive				
Streptococcus pneumoniae	12	9	7	2
Group A/C/G streptococci	8	6	11	1
Group B streptococci	1	4	2	0
Enterococci	2	1	2	0
Staphylococcus aureus	25	19	25	2
Coagulase negative Staphylococcus	7	2	1	1
Staphylococcus lugdunensis	1	0	0	0
Staphylococcus epidermidis	3	4	2	0
Staphylococcus saprophyticus	1	0	0	0
Non haemolytic streptococcus	0	1	1	0
Gram-negative				
Escherichia coli	46	44	37	3
ESBL - Escherichia coli	1	0	2	0
Klebsiella spp.	12	8	7	0
Other Enterobacteriaceae	9	2	8	1
Moraxella catarrhalis	6	1	4	1
Pseudomonas aeruginosa	4	3	4	0
Haemophilus spp.	16	9	10	1
Campylobacter spp.	1	0		0
Legionella spp.	0	0	4	0
Unspecified gram-negative rods	4	1	4	0
Anaerobic bacteria				
Clostridium spp.	2	0	4	0
Bacteroides spp.	0	0	0	0
Unspecified gram-positive rods	2	1	1	0
Other				
Mycoplasma pneumoniae	1	2	0	0
Mycobacterium tuberculosis	0	1	0	0
Candida spp.	4	1	1	1
H1N1	0	0	1	0
Herpesvirus	1	0	0	0
Sapovirus	0	1	0	0
VZV	0	0	1	0
Norovirus	0	0	1	0

^*^The added number of pathogens exceeds the number of cases because one patient could have more than one pathogen found in obtained cultures

Among gram-positive microorganisms *Staphylococcus aureus* was found in 71 cases (16% of all positive tests) and *Streptococcus pneumoniae* in 30 cases (7% of all positive tests). As for gram-negative microorganisms *Escherichia coli* accounted for almost one-third of the isolates (130 cases – 30% of all positive tests). Anaerobes were isolated in 10 cases (2% of all positive tests). Fungal infection due to *Candida* spp. were found in 7 (1.6% of all positive tests) cases. There were 3 cases (0.7% of all positive tests) with ESBL (all *Escherichia coli*), found in urine in patients with urinary tract infections.

*Haemophilus influenzae* was the most common pathogen found in cases with lower respiratory tract infections, followed by *Streptococcus pneumoniae*. The most frequent pathogen in urinary tract infections was *Escherichia coli*. The most common pathogen in skin-soft tissue infections was *Staphylococcus aureus.*

By site of infection, the most common focus of infection was lower respiratory tract (407 cases, 39% of all infections), urinary tract (204 cases, 19% of all infections), followed by the skin, soft tissue, and bones (99 cases, 9% of all infections). These three major sites accounted for more than 67% of all sources of infection. Two or more sites were involved in 87 cases (8% of all infections) of community-acquired infections. Women were admitted with bronchitis and urinary tract infections more often, while men had more catheter related infections. The primary focus of infection was unknown in 274 cases (26% of all infections).

In 427 cases (40% of all cases with infection) the focus of infection was found by imaging techniques, in 275 cases (26% of all cases with infection) by finding the responsible pathogen, and in 29 cases (2.8% of all cases with infection) by both. Of all radiological analyses with positive radiological findings suggesting infection 87% were chest x-rays. In 58 cases (6% of all cases with infection) no microbiological analyses were performed. In 27 cases (3% of all cases with infection) neither microbiological analyses nor imaging or other diagnostic methods were performed. In this group 4 cases were treated for recurrent, previously diagnosed infections, 6 were diagnosed by examining the patients clinically, and in 16 cases infection diagnosis remained uncertain.

Positive blood cultures were found in 99 cases (Table 4). Twelve cases had bacteremia without SIRS. In 76 patients a single bacteremia episode was found whereas 10 patients experienced multiple episodes of bacteremia. Of those patients, 8 had repeatedly positive blood cultures with the same pathogen, most commonly *Staphylococcus aureus* (10 episodes in 4 patients) and *Escherichia coli* (7 episodes in 3 patients). Two other patients had consecutive episodes of bacteremia with different species. The most frequent pathogens involved in the bacteremia were: *Escherichia coli* (47%), *Staphylococcus aureus* (19%) and *Klebsiella* spp. (12%). Bacteremia was more frequent in patients with diabetes (20/86 patients (23%)) and connective tissue disease (18/86 patients (21%)) in men (47 patients (55%)), and older population (mean age 70). There were 18 cases (18%) of patients with cancer who had positive blood cultures. The use of immunosuppressive medications was found in 25 patients or in 29 cases (29%) with bloodstream infections.

**Table 4:** Cases with bloodstream infection related to infection severity

Microorganism	Infection without SIRS	Sepsis	Severe sepsis	Septic shock
Gram-positive				
Streptococcus pneumoniae	1	1	3	2
Group A/C/G streptococci	0	0	2	1
Group B streptococci	0	1	2	0
Enterococcus spp.	0	0	2	0
Staphylococcus aureus	2	4	13	0
Staphylococcus lugdunensis	1	0	0	0
Staphylococcus epidermidis	0	2	2	0
Non haemolytic streptococcus	0	1	1	0
Gram-negative				
Escherichia coli	5	22	17	3
Klebsiella spp.	2	4	6	0
Other Enterobacteriaceae	0	0	2	0
Pseudomonas aeruginosa	1	0	0	0
Haemophilus spp.	0	0	1	0
Anaerobic bacteria				
Clostridium spp.	0	0	1	0

^*^The added number of pathogens exceeds the number of cases because one patient could have more than one pathogen found in blood cultures

By site of infection, most patients with bacteremia had either pneumonia or urinary tract infections.

## Discussion

### Principal findings

Infection was documented in 74% of the patients. The most common focus of infection was lower respiratory tract infection, followed by urinary tract infection and skin-soft tissue/bone-joint infections. Gram-negative pathogens, especially *Escherichia coli*, predominated. The most frequent pathogen was *Haemophilus influenzae* in lower respiratory tract infections, *Escherichia coli* in urinary tract infections and *Staphylococcus aureus* in skin-soft tissue infections. In the group with bacteremia urinary tract and lower respiratory tract foci predominated. Among those with bacteremia, *Escherichia coli* and *Staphylococcus aureus* were the most frequent pathogens.

### Comparison to other studies

#### Community-acquired infections

To the best of our knowledge there are very few studies on hospitalized patients with community-acquired infection of any severity and with different foci of infection. Most of existent studies focus either on more severe forms of infection, mostly severe sepsis and septic shock [18–20]; on particular infections such as pneumonia [21, 22], urinary tract infections [23, 24]; on a specific pathogen [25]; on specific aspect like antibiotic consumption [26], or on specific gender/gender dependent differences [27, 28]. According to Alberti et al. differences in studies´ definitions and population make it difficult, and to compare their findings [29]. This is why we used “infection-approach” focusing on infection rather than only on sepsis, and analyzing data associated with community-acquired infections of any severity in adults. To our knowledge the only study addressing similar aspects of community-acquired infection/sepsis was the Danish study by Ostrowski et al. [30].

### Microbiology

We found that gram-negative infections were documented in more than a half of the cases with identified pathogen. This is similar to the study by Henriksen at al. [31]. Flaatten et al. found gram-negative pathogens in 57% of cases in the study population that consisted of both community-acquired and hospital-acquired infections [32]. Søgaard et al. found gram-negative pathogens in 51% of cases with bloodstream infections [33]. Martin et al. showed predominance of gram-positive pathogens [34]. The EPIC II study analyzed surgical and medical patients admitted to the Intensive Care Unit with both community-acquired infection and hospital-acquired infections, and found more gram-negative pathogens [35]. Predominance of gram-positive pathogens in the blood were found in studies by the SepNet Critical Care Trials Group and Karlsson [20, 36]. Vincent et al. found almost equal rate of gram-positive and gram-negative bacteria, with slight predominance of gram-positive pathogens in their study of medical and surgical patients with sepsis admitted in 198 Intensive Care Units in 24 European countries [37], and similar results were found in two Spanish studies from Blanco et al. [38] and Esteban et al. [39]. Sands found that 40% of all episodes with sepsis syndrome had gram-negative pathogens (Enterobacteriaceae) [40]. In this study gram-negative pathogens were mostly found in all sepsis syndrome cases with exclusion of cases with bacteremia, where gram-positive pathogens were mostly found, with *Staphylococcus aureus* as the most frequent. *Escherichia coli*, *Klebsiella* spp. and *Staphylococcus aureus* were the most frequent pathogens in a Korean study of medical and surgical patients admitted to the Emergency Department and Intensive Care Units by Park et al. [41]. In a Columbian study Rodríguez at al. showed that *Escherichia coli*, *Staphylococcus aureus* and *Klebsiella* spp. were the most frequent pathogens found in the blood as shown in our study [42].

### Focus of infection

The most frequent focus of infection in our study, in all groups, were lower respiratory tract, urinary tract and skin-soft tissue/bone-joint infections, which is consistent with other studies focusing on community-acquired infections [30, 31, 42, 43]. Lower respiratory tract infections were the focus in almost 50% of all infections among patients with severe sepsis or septic shock. This too is consistent with the study by Ostrowski et al. and Henriksen et al. [30, 31]. We found that only 3% of cases had an abdominal focus of infection, probably reflecting that our study did not include patients admitted to the Surgical Department. Other studies showed higher percentage of abdominal infections [20, 26, 29, 31, 37, 38]. All these studies, with exception of the first, involved surgical patients and/or patients with hospital-acquired infection.

We could not determine the focus of infection in 26% of the cases with clinical infection according to our study entry criteria. In the group of sepsis of any severity 25% of cases had undetermined focus of infection. Wang et al. showed that 17% of their cases with community-acquired sepsis were undetermined, with extra 2% of cases with fever of unknown origin [44]. The occurrence of undetermined infection foci will be discussed in the study limitation section of this article.

### Strengths

This is the first study on etiology and focus of infection in the context of community-acquired infections requiring hospitalization in the Faroe Islands. This is a prospective, observational study conducted during a period of 18 months. In many circumstances observational data provide the only evidence to guide future management [45]. Our study probably describes a more accurate real-life picture of the infectious diseases panorama among hospitalized patients with community-acquired infections.

### Limitations

This study included only patients admitted and treated at the Department of Medicine and medical patients admitted at the Intensive Care Unit at the National Hospital of the Faroe Islands, thus not making this a nation-wide study. However, according to our experience, most patients with community-acquired infections are admitted at the Department of Medicine and according to Statistics Faroe Islands (Hagstova Føroya) [11], the National hospital of the Faroe Islands has a catchment area of 80% which could argue that we showed nationally representative data. Another limitation of the study is that it is likely that some patients with abdominal and gynecological focus of infection would be diagnosed and treated at the Surgical Department of the hospital. This selection bias would underestimate the frequency of abdominal, gynecological and other surgical infections. Another limitation is that we may have underestimated the number of patients with community-acquired infection as some patients admitted with noninfectious diseases had an infection at the time of admission. These patients were not always fully investigated for infection in the first 48 h. The focus of infection or a plausible pathogen was not identified in all patients. An explanation could be that some patients were already in antibiotic treatment prior to hospitalization and diagnostic sampling. This, according to other studies, can influence the diagnostic processes [46]. Even though we found that in most cases proper diagnostic tests were done, diagnostic workup may have been insufficient or incomplete in some cases since all decisions concerning diagnostics and treatment were up to the treating physician. This may also have contributed to the number of cases with unidentified focus and lack of proven microbiological etiology.

## Conclusion

This is the first study done in the Faroe Islands focusing on etiology and focus of infection in patients with community-acquired infections requiring hospitalization. The lower respiratory tract and urinary tract were the most site of infection. The most common etiologic agents were gram-negative pathogens.

### Additional file

Additional file 1: Pre-study defined consensus definitions. (DOCX 18 kb)

## Appendix 1

**Table 5:** Modified SOFA score

Organ system	Variable	0	1	2	3	4
Respiratory	PaO2/FiO2 or O2 saturation	≥400 or ≥ 98%	< 400 or 97–90%	< 300 or 89–80%	< 200 with respiratory support or < 79%	< 100 with respiratory support
Coagulation	Platelets (10^9^/l)	≥150	< 150	< 100	< 50	< 20
Liver	Bilirubin (μmol/l)	< 20	20–32	33–101	102–204	> 204
Cardiovascular	Hypotension	≤70	≤70 fluid only	≤70*	≤70**	≤70***
Brain	Glasgow Coma Scale	15≤	13 and 14	10–12	6–9	< 6
Kidney	Creatinine (μmol/l)	< 110	110–170	171–299	300–440	> 440

*Modification (inclusion of Oxygen saturation as variable for respiratory failure)

*Dopamine ≤5 μg/kg min for at least 1 h or Dobutamine (any dose)

**Dopamine > 5 μg/kg min or Epinephrine ≤0,1 μg/kg min or Norepinephrine ≤0,1 μg/kg min

***Dopamine > 15 μg/kg min Epinephrine > 0,1 μg/kg min or Norepinephrine > 0,1 μg/kg min

## Appendix 2

Organ dysfunction criteria:

1. Glasgow coma scale of less than or equal to 14
2. PaO2 of less than or equal to 9,75 kPa
3. Oxygen saturation of less than or equal to 92%
4. PaO2/FiO2 of less than or equal to 250
5. Systolic blood pressure of less than or equal to 90 mmHg
6. Systolic blood pressure decrease of more than or equal to 40 mmHg
7. pH of less than or equal to 7,3
8. Lactate of more than or equal to 2,5 mmol/l
9. Creatinine of more than or equal to 177 μmol/l
10. 100% increase of creatinine in patients with known kidney disease
11. Oliguria of less than or equal to 30 ml/h in more than 3 h or less than or equal to 0,7 l/24 h
12. International normalized ratio of higher than or equal to 1,5
13. Platelets of less than or equal to 100x10π/l
14. Bilirubin of more than or equal to 43 μmol/l
15. Paralytic ileus

## Abbreviations

ESBL: Extended-Spectrum Beta-Lactamase Producing Bacteria
GCS: Glasgow Coma Scale
qSOFA: quick Sepsis Related Organ Failure Assessment
SIRS: Systemic Inflammatory Response Syndrome
SOFA: Sepsis-related Organ Failure Assessment
