# Supplementary material for: Focus of infection and microbiological etiology in community-acquired infections in hospitalized adult patients in the Faroe Islands
Source: BMC Infect Dis. 2019 Jan 7;19:16. doi: 10.1186/s12879-018-3650-3 (PMC6322335; doi:10.1186/s12879-018-3650-3)
Supplement: Supplementary file 1 — Pre-study defined consensus definitions. (DOCX 18 kb) [file 12879_2018_3650_MOESM1_ESM.docx]

# Additional file 1.

## Pre-study defined consensus definitions:

### Meningitis:

- Symptoms and signs: (Headache, fever, neck stiffness, altered sensorium)
- Pleocytosis CSF (> 5 cells/microl)
- +/- relevant microbiology (incl. positive blood cultures), PCR - CSF

### Encephalitis:

- altered levels of consciousness
- pleocytosis In CSF (> 5 cells/microl)
- +/- relevant microbiology, PCR – CSF
- +/- radiology
- +/- EEG
- At least 1 of 3 diagnostic methods should be consistent with encephalitis

### Upper respiratory infections:

- Otitis:

Symptoms and signs

- - Objective otoscopic findings
  - +/- relevant microbiology from ear swab, puss, liquid from ear or from paracentesis
- Sinusitis:
  - Symptoms and signs
  - Radiology
  - +/- relevant microbiology from nasal swab
- Tonsillitis:
  - Symptoms and signs
  - +/- relevant microbiology from throat swab
- Laryngitis
  - relevant microbiology from throat swab

### Lower respiratory infections:

- Bronchitis:
  - Symptoms and signs
  - + relevant microbiology form secretions from respiratory system (sputum, tracheal aspirate, BAL)
  - +/- positive blood cultures
  - - infiltrate
- Pneumonia:
  - Symptoms and signs
  - Radiology
  - +/- relevant microbiology, PCR form secretions from respiratory system (sputum, tracheal aspirate, BAL)
  - +/- positive blood cultures

### Endocarditis:

- + echocardiography or Duke criteria
- +/- relevant microbiology (incl. positive blood cultures)

### Cholangitis:

- Blood biochemistry
- + radiology
- +/- relevant microbiology (incl. positive blood cultures)

### Cholecystitis:

- Blood biochemistry
- + radiology
- +/- relevant microbiology (incl. positive blood cultures)

### Gastroenteritis:

- Symptoms and signs
- + relevant microbiology form feces; (incl. positive blood cultures)

### Peritonitis:

- Symptoms and signs
- + radiology or purulent ascites fluid
- +/- relevant microbiology from peritoneal fluid
- +/- positive blood cultures

### Peritonitis (peritoneal dialysis patients)

- Abdominal pain
- + Combur test
- +/- relevant microbiology from peritoneal fluid
- +/- positive blood cultures

### Cystitis:

- Symptoms and signs: dysuria
- + dip test
- +/- relevant microbiology from urine
- +/- positive blood cultures

### Pyelonephritis:

- Symptoms and signs: dysuria + pain in the flank
- + dip test
- +/- relevant microbiology from urine
- +/- positive blood cultures

### Female genital tract infection:

- Symptoms and signs
- Gynecological findings
- +/- relevant microbiology from swabs taken from vagina or cervix
- +/- positive blood cultures

### Skin and soft tissue infections:

- Symptoms and signs (wound/infected wound, skin changes)
- +/- relevant microbiology from wound swabs
- +/- positive blood cultures

### Bone/joint infection:

- Symptoms and signs
- + image diagnotics
- +/- relevant microbiologiy from joint aspirate or bone biopsies

### Catheter infection:

- Symptoms and signs
- +/- radiology
- + relevant microbiology from the skin around catheter or from catheter tip swab
- +/- positive blood cultures

### Group I:

- Symptoms and signs
- + culture / PCR form usually sterile sites except urine

### Group II:

- Symptoms and signs
- + finding by imaging, histology, cytology or serology

### Uncertain group:

Infection signs are present without confirmed infection
